# Feasibility of comprehensive genomic profiling using endoscopic ultrasound‐guided tissue acquisition with a 22‐gauge Franseen needle

**DOI:** 10.1002/deo2.365

**Published:** 2024-04-15

**Authors:** Kazunaga Ishigaki, Yousuke Nakai, Go Endo, Kohei Kurihara, Kota Ishida, Shuichi Tange, Rintaro Fukuda, Shinya Takaoka, Yurie Tokito, Yukari Suzuki, Hiroki Oyama, Sachiko Kanai, Tatsunori Suzuki, Tatsuya Sato, Ryunosuke Hakuta, Tomotaka Saito, Tsuyoshi Hamada, Naminatsu Takahara, Aya Shinozaki‐Ushiku, Mitsuhiro Fujishiro

**Affiliations:** ^1^ Department of Gastroenterology Graduate School of Medicine The University of Tokyo Tokyo Japan; ^2^ Department of Chemotherapy The University of Tokyo Hospital Tokyo Japan; ^3^ Department of Endoscopy and Endoscopic Surgery The University of Tokyo Hospital Tokyo Japan; ^4^ Department of Pathology The University of Tokyo Hospital Tokyo Japan

**Keywords:** comprehensive genomic profiling, endoscopic ultrasound‐guided fine needle aspiration, Franseen needle, image analysis, pancreatobiliary cancer

## Abstract

**Aim:**

Comprehensive genomic profiling (CGP) test for solid tumors is now increasingly utilized in clinical practice, especially in pancreatobiliary cancer, and specimens obtained by endoscopic ultrasound‐guided tissue acquisition (EUS‐TA) are often submitted for tissue‐based CGP test. In this study, we evaluated the feasibility of EUS‐TA using a 22‐gauge Franseen needle for the CGP test.

**Methods:**

Consecutive patients with solid tumors who underwent EUS‐TA using a 22‐gauge Franseen needle, and whose tissue samples were pre‐checked for suitability for CGP test, were included in this single‐center, retrospective analysis. The success rates of appropriate sample collection for CGP evaluated by pathologists (1st quality control) and CGP test (2nd quality control) were evaluated. In addition, The EUS‐TA slides were evaluated for the tissue area and tumor area content, using the image software.

**Results:**

A total of 50 cases, with 78% of pancreatic cancer, were included in the analysis. A median of 3 passes of EUS‐TA were performed with an adverse event rate of 4%. The success rates for 1st and 2nd quality control for CGP tests were 86% and 76%, respectively. The image analyses suggested EUS‐TA specimen did not always fulfill CGP test criteria, with 18% of tissue area ≥16 mm^2^ and 38% of tumor area content ≥20%, even in cases with successful CGP tests. The suction method yielded a significantly larger amount of DNA but without a significant difference in the multivariate analysis.

**Conclusions:**

The present study demonstrated the feasibility of EUS‐TA using a 22‐gauge Franseen needle for CGP test.

## INTRODUCTION

Comprehensive genomic profiling (CGP) tests for solid tumors have rapidly gained popularity.^1^, ^2^, ^3^, ^4^, ^5^, ^6^, ^7^ Although the ratio of druggable mutations diagnosed in CGP tests is still small, some reports showed an improved prognosis when treatment based on genomic abnormalities was available.^8^, ^9^ As limited treatment options of conventional cytotoxic chemotherapeutic agents are currently available for unresectable pancreatobiliary cancers, many patients with pancreatobiliary cancers undergo CGP testing to expand their treatment options such as *BRCA1/2* in pancreatic cancer,^10^ *FGFR2* fusion in biliary tract cancer,^11^, ^12^ and microsatellite instability‐high and tumor mutational burden‐high in solid tumors.^13^

The CGP test can be performed using tissue samples, or blood samples when tissue samples are unavailable. The tissue‐based CGP test is considered more sensitive because circulating tumor DNA may not be detected by a blood‐based CGP test in some cases.^14^ However, the CGP test often needs to be performed using non‐surgically collected small tissue samples, especially in cases with advanced pancreatobiliary cancers.

Endoscopic ultrasound‐guided tissue sampling (EUS‐TA) is an established procedure for pathological diagnoses of pancreatic tumors,^15^, ^16^, ^17^, ^18^ subepithelial lesions, and lymph node metastases; however, the reported success rates of CGP analysis using EUS‐TA vary from 57.4% to 97.0%.^19^, ^20^, ^21^, ^22^, ^23^, ^24^, ^25^ Furthermore, there is no consensus regarding the best technique of EUS‐TA for CGP testing. Although high success rates for the CGP test were reported using 19‐gauge needles,^24^, ^25^ their maneuverability is poor and the incidence of adverse events (AEs) is high, which may discourage the use of 19‐gauge needles outside high‐volume centers. Meanwhile, EUS‐TA using a 22‐gauge fine‐needle biopsy (FNB) needle can be widely accepted as a standard of care in most centers but its usefulness for CGP test has not been fully elucidated. Thus, in this study, we evaluated the feasibility of CGP tests using EUS‐TA with a 22‐gauge Franseen needle.

## METHODS

### Patients

This is a single‐center, retrospective study at the University of Tokyo Hospital. Consecutive patients whose EUS‐TA samples using a 22‐gauge Franseen needle were evaluated for CGP tests between April 2018 and November 2022 were included in the analysis. Written informed consent for the procedure was obtained from all patients, and consent for using the retrospective data for research was obtained on an opt‐out basis. This study was approved by the Research Ethics Committee of the Faculty of Medicine, University of Tokyo (No. 20585).

### EUS‐TA

A 22‐gauge Franseen needle (Acquire; Boston Scientific Japan) was advanced into the target lesion under EUS guidance, and 20–40 to‐and‐fro movements were performed within the target lesion. In the suction technique, 10–20 cc of suction was applied with a syringe, and in the slow‐pull technique,^26^ minimum negative pressure was applied by pulling the needle stylet slowly and continuously. In the no‐suction technique, suction using a syringe or a stylet pull was not applied. The obtained material was expressed entirely on a glass slide or in a formalin bottle by reinserting the stylet or flushing with air or saline. The number of passes was decided at the discretion of the attending physician; in general, EUS‐TA was repeated until a sufficiently visible core tissue was obtained. Rapid on‐site evaluation (ROSE) was not routinely performed due to the limited availability in our center. Antithrombotic agents were administered or stopped according to the Japan Gastroenterological Endoscopy Society guidelines for gastroenterological endoscopy in patients undergoing antithrombotic treatment.^27^ Tissues collected using EUS‐TA were promptly immersed in a 10% neutral‐buffered formalin solution. Formalin fixation was performed for 24 h, and all specimens obtained by multiple punctures were combined to create a single Formalin‐fixed paraffin‐embedded (FFPE) block, which was used for pathological diagnosis and CGP testing.

### CGP analysis

First, hematoxylin and eosin (HE)‐stained slides prepared from FFPE specimens were pre‐checked by pathologists based on appropriate specimen criteria, including tumor cell content and tissue area evaluated for the panel to be submitted (1st quality control [QC] data). Two CGP tests by tissue samples were used; OncoGuide NCC Oncopanel System (NOP) and FoundationOne CDx (F1CDx), with 124 genes measured in NOP and 324 in F1CDx. In NOP, a control blood sample was also obtained, allowing accurate germline mutations. Furthermore, NOP provides data on DNA yield (2nd QC data). When 1st and 2nd QC failed and repeated tissue sampling was difficult, the CGP test was performed using FoundationOne Liquid CDx (F1 liquid).

### Outcomes

Cases for which pre‐checks were conducted by pathologists with the intention of submitting CGP tests were defined as the intention‐to‐treat cohort, while cases that passed the pre‐checks and were actually submitted for CGP tests were defined as the per‐protocol (PP) cohort. The primary endpoint was the 2nd QC success rate in an intention‐to‐treat analysis. The secondary endpoints were as follows: AEs of EUS‐TA, appropriate sample collection rate (1st QC success rate), 2nd QC success rate in a PP analysis, DNA yield of 2nd QC, success rate of CGP test including tissue and liquid‐based panel, frequency of druggable gene mutations, the rate of patients who received matched treatment, and tumor area content and tissue area measured by the ImageJ.^28^ The appropriate sample collection rate was defined as the success rate of the pre‐check performed by pathologists. A successful CGP test was defined as successful sequencing irrespective of disclosure of the CGP test results to patients. For cases submitted to F1CDx, we defined the quality of the specimen as “Pass” or “Qualified” to indicate “Success”. Similarly, for cases tested on the NOP, disclosing results within the reference range was also considered “Success”.

### Image analysis of EUS‐TA specimen by ImageJ

**FIGURE 1:**
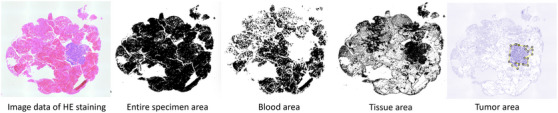
Image processing with ImageJ. (a) Image of hematoxylin and eosin staining. (b) The entire specimen area on ImageJ. (c) Blood area on ImageJ. (d) Tissue area on ImageJ. (e) Tumor area on ImageJ.

ImageJ is a Java‐based image processing program developed at the National Institutes of Health, which allows the quantification of images of pathological specimens. Photographs of HE‐stained specimen slides collected by EUS‐TA were imported as digital data, and the area of the entire specimen and blood area were calculated by extracting specific colors from the image data. The tissue area was defined as the area of the entire specimen excluding the blood area. The tumor area was calculated by grossly identifying the tumor cells and manually extracting the tumor area. Tumor area content was defined as tumor area divided by tissue area (Figure 1).

### Statistical analysis

Continuous variables were presented as medians and ranges, and categorical variables as numbers and percentages. Statistical comparisons were performed using the Mann–Whitney U test for continuous variables and the chi‐square test or Fisher's exact test for categorical variables. Factors affecting the successful CGP test were evaluated using the logistic regression analysis. Factors affecting DNA yield were evaluated using the multivariate regression analysis in cases with disclosed 2nd QC data of NOC. Statistical significance was set at *p* < 0.05. The R software (version 2.14.0; R Development Core Team: http://www.r‐project.org) was used for all statistical analyses.

## RESULTS

### Patient characteristics and EUS‐TA

Patient characteristics are shown in Table 1. A total of 50 patients to whom the CGP test was attempted using EUS‐TA samples obtained by a 22‐gauge Franseen needle were included in the analysis. There were 25 male patients (50%) with a median age of 70 (range, 42–88) years. Pancreatic cancer was the most common disease (39 patients, 78%).

**TABLE 1:** Patient characteristics.

Sex, male	25 (50)
Age, years	70 (42–88)
Final diagnosis	
Pancreatic cancer	39 (78)
Gallbladder cancer	5 (10)
Hilar cholangiocarcinoma	2 (4)
Intrahepatic cholangiocarcinoma	1 (2)
Neuroendocrine carcinoma	1 (2)
Combined hepatocellular‐cholangiocarcinoma	1 (2)
Cancer of unknown primary	1 (2)

*Note*: Numbers are shown in *n* (%) or median (range).

 Details of EUS‐TA are shown in Table 2. EUS‐TA was performed with suction in 36 (72%), without suction in 12 (24%), and with slow pull in two (4%). The major target lesions of EUS‐TA were located in the body or tail of the pancreas in 23 (56%) and the head of the pancreas in 14 (28%). The median number of punctures was 3 (range, 2–7). Early AEs were observed in 4%; one mild pancreatitis and one mild hemorrhage.

**TABLE 2:** Detail of endoscopic procedures and comprehensive genomic profiling (CGP) test.

Location of the lesion	
Pancreas head	14 (28)
Pancreas body or tail	23 (46)
Lymph node	10 (20)
Liver	3 (6)
Tumor size, mm	30 (12–80)
Suction method	
Suction/no suction/slow‐pull	36 (72)/12 (24)/2 (4)
Number of passes	3 (2–7)
Rapid on‐site evaluation	3 (6)
Procedure time, minutes	35 (18–86)
Endoscopist, experts	15 (30)
CGP test submitted for pre‐check, NOP/F1CDx	48/2

*Note*: Numbers are shown in *n* (%) or median (range).

Abbreviations: CGP, comprehensive genomic profiling; F1CDx, FoundationOne CDx; NOP, OncoGuide NCC Oncopanel System.

### CGP test results

**FIGURE 2:**
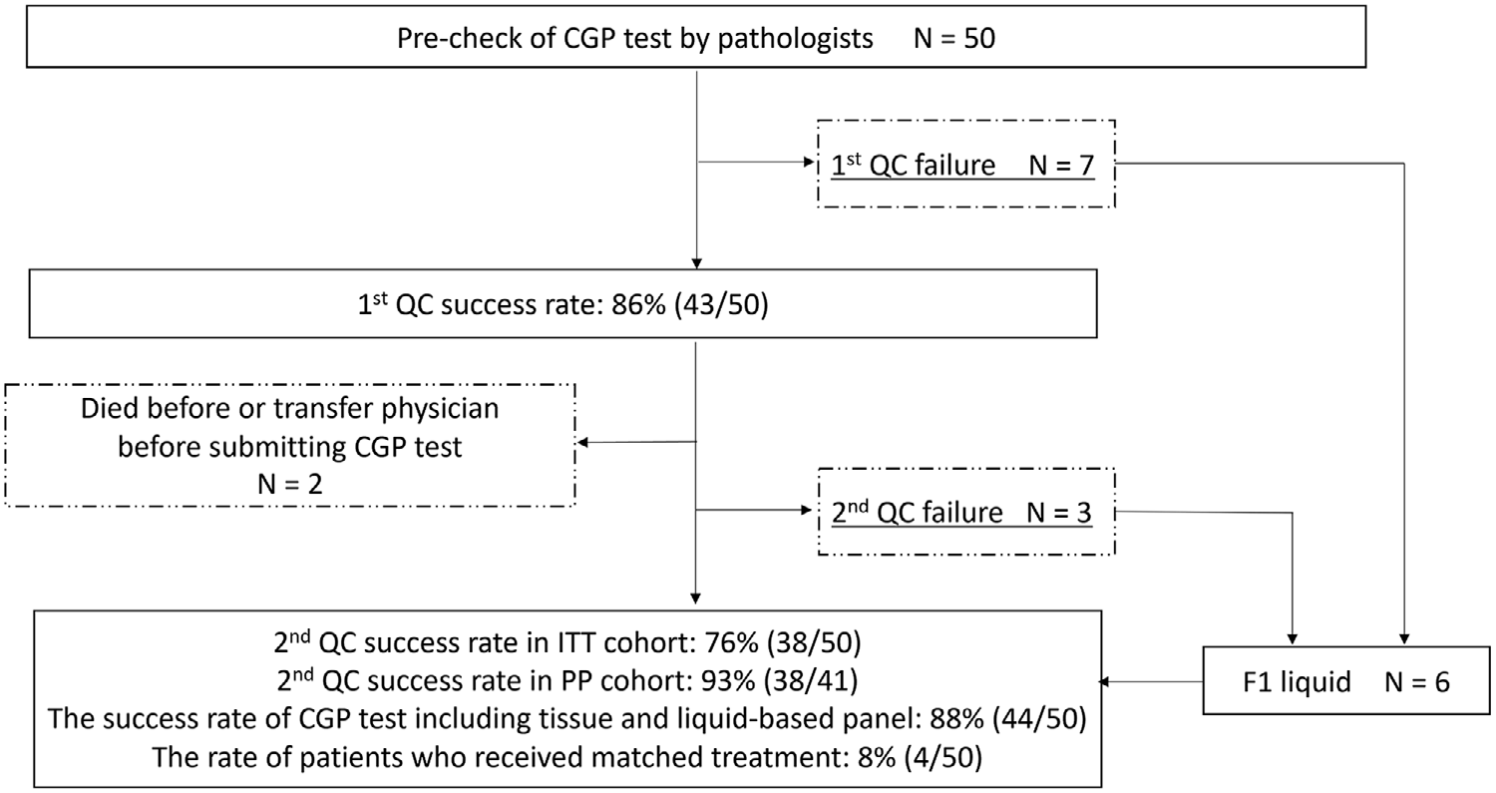
Flowchart. CGP, comprehensive genomic profiling; QC, quality check; F1 liquid, FoundationOne Liquid CDx.

A flowchart and results of the CGP test are presented in Figure 2 and Table 3, respectively. The attempted CGP tests for submission were 48 cases for NOP and two cases for F1CDx. EUS‐TA specimens passed a pre‐check of the CGP test in 43 patients, with 1st QC success rate of 86%. The reasons for 1st QC failure were insufficient tissue sample volume in four, insufficient tumor cellularity in two, and no malignancy in one. After a successful 1st QC, two patients died before submission of the GCP test, and 41 patients underwent the CGP test. Finally, the 2nd QC success rate was 76% (38/50) in an intention‐to‐treat analysis. In cases other than pancreatic cancer, successful CGP testing was achieved in all cases. CGP tests using F1 liquid were performed in three out of seven of the 1st QC failures and three out of three of the 2nd QC failures, and the success rate of CGP tests including tissue and liquid‐based panels was 88% (44/50). Druggable gene mutations were found in 10 patients (20%), and four patients (8%) underwent matched treatment (Table S1).

**TABLE 3:** Results of comprehensive genomic profiling (CGP) test.

		Type of CGP tests		Primary cancer	
	Total (*n* = 50)	NOP (*n* = 48)	F1CDx (*n* = 2)	Pancreas (*n* = 39)	Non‐pancreas (*n* = 11)
The success of 1st QC (pre‐check by pathologists)	43 (86)	41 (85)	2 (100)	32 (82)	11 (100)
Death before submission of CGP test	2 (4)	2 (4)	0 (0)	2 (5)	0 (0)
Submission of CGP test, *n* (%)	41 (82)	3 (6)	2 (100)	30 (77)	11 (100)
Suspension of CGP test due to 2nd QC failure	3 (6)	3 (6)	0 (0)	3 (8)	0 (0)
Success of 2nd QC (intention‐to‐treat)	38 (76)	36 (75)	2 (100)	27 (69)	11 (100)
The success of 2nd QC (per protocol, among 41 CGP submissions)	38 (93)	36 (75)	2 (100)	27 (90)	11 (100)

*Note*: Numbers are shown in *n* (%).

CGP, comprehensive genomic profiling; F1CDx, FoundationOne CDx; NOP, OncoGuide NCC Oncopanel System; QC, quality check.

### Second QC data of DNA yield in NOP

**FIGURE 3:**
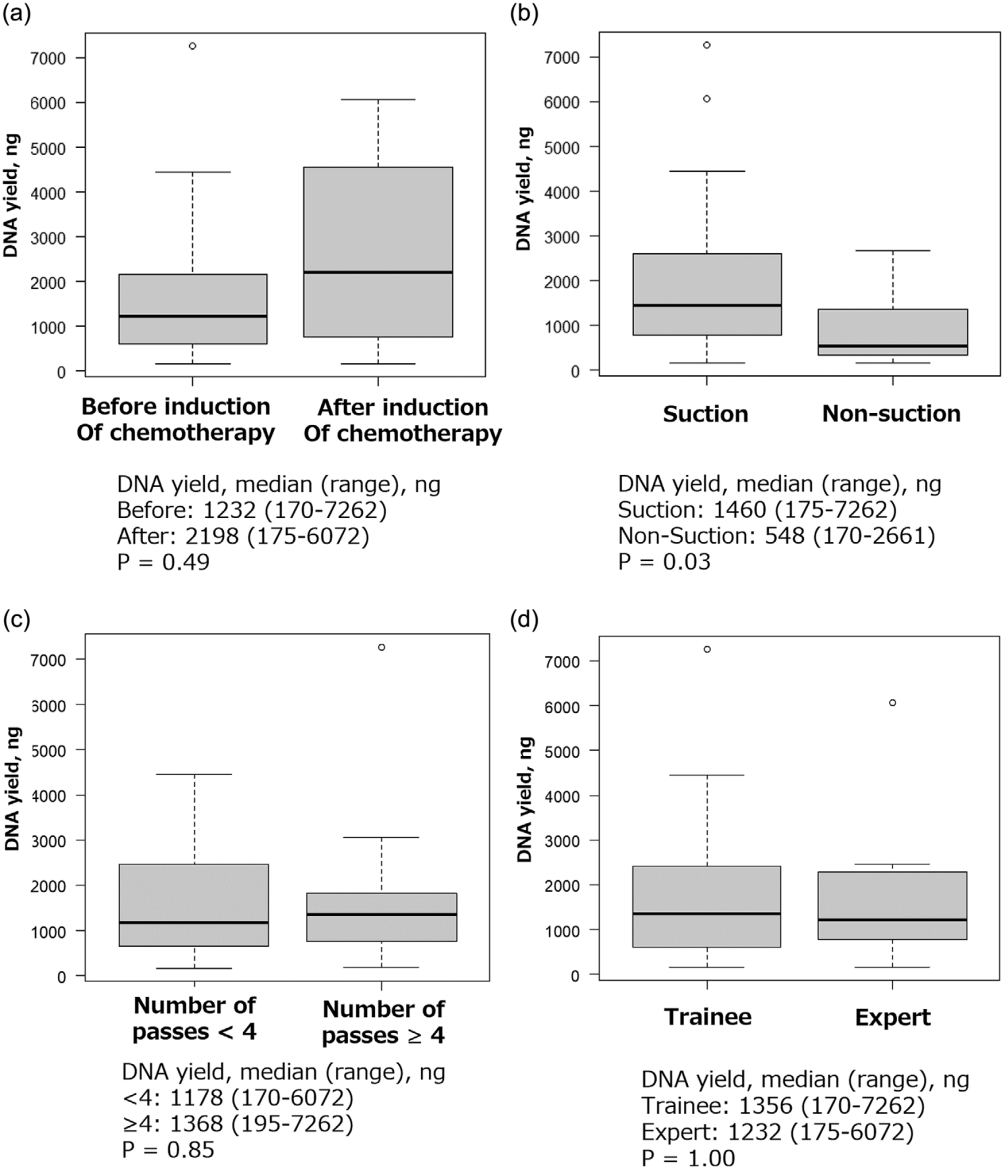
Comparison of DNA yield. (a) Timing of endoscopic ultrasound‐guided tissue acquisition (EUS‐TA); Median 1232 (range, 170–7262) and 2198 (range, 175–6072) ng in cases with EUS‐TA before and after induction of chemotherapy (*p* = 0.49). (b) Suction methods; Median 1460 (range, 175–7262) and 548 (range, 170–2661) ng in cases with suction and no suction methods (*p* = 0.03). (c) Number of passes; Median 1178 (range, 170–6072) and 1368 (range, 195–7262) ng in cases with the number of passes <4 and ≥4 (*p* = 0.85). (d) Endoscopist experiences; Median 1356 (range, 170–7262) and 1232 (range, 175–6072) ng by trainees and experts (*p* = 1.00).

The results of the 2nd QC data are shown in Figure 3. In 36 cases undergoing NOP, the median DNA yield was 1343 (range, 170–7262) ng. Figure 3 shows the DNA yield according to the timing and techniques of EUS‐TA and endoscopist experiences. The suction method yielded a significantly larger amount of DNA with a median of 1460 and 548 ng in cases with and without suction methods (*p* = 0.03), but the difference was not statistically significant in the multivariate analysis (Table 4).

**TABLE 4:** Multivariate analysis of factors related to DNA yield.

	Regression coefficient estimates (95%CI)	SE	T statistic	*p*
EUS‐TA before chemotherapy introduction	−1012 (−2674–650)	818	−1.24	0.22
Method of suction, suction	1051 (−70–2172)	552	1.90	0.07
Number of passes ≥4	91 (−960–1142)	517	0.18	0.86
Endoscopist, Expert	105 (−1023–1233)	555	0.19	0.85

Abbreviations: CI, confidence Interval; EUS‐TA, endoscopic ultrasound‐guided tissue sampling; ROSE, rapid on‐site evaluation; SE, standard Error.

### Image analysis of EUS‐TA samples by ImageJ

Slides of EUS‐TA specimens were evaluated using the image software, ImageJ, to explore the association of successful CGP test with tissue area and tumor area content on EUS‐TA slides (Table 5). The median tissue area and tumor area content was 7.1 (range, 0.1–49.8) mm^2^ and 17% (range, 0–59%), respectively. According to the NOP specimen requirements, nine (18%) had ≥16 mm^2^ tissue area and 19 patients (38%) had ≥20% tumor area content. Only four patients (8%) had both tissue area of ≥16 mm^2^ and tumor area content of ≥20%.

**TABLE 5:** Tissue area and tumor area content on ImageJ analysis.

Tissue area, mm^2^	7.1 (0.1–49.8)
Tumor area content, %	17 (0–59)
Tissue area ≥16 mm^2^	9 (18)
Tissue area ≥25 mm^2^	4 (8)
Tumor area content ≥20%	19 (38)
Tissue area ≥16 mm^2^ and tumor area content ≥20 %	4 (8)
Tissue area ≥25 mm^2^ and tumor area content ≥20 %	1 (2)

*Note*: Numbers are shown in *n* (%) or median (range).

Prognostic factor analyses revealed that non‐pancreatic cancer and tumor cellularity ≥20% on pre‐check by pathologists were as potentially favorable factors for successful CGP tests. Meanwhile, if the tissue area was ≥16 mm^2^ on ImageJ, CGP tests were successful in all cases. (Table 6).

**TABLE 6:** Factors related to successful comprehensive genomic profiling (CGP) test.

	Success (*n* = 38)	Failure (*n* = 12)	*p*
Age, years	69 (42–88)	72 (44–80)	0.99
Sex, male	20 (53)	5 (42)	0.74
Tumor size, mm	30 (12–80)	31 (13–64)	0.61
Primary tumor, pancreas	27 (71)	12 (100)	0.05
Method of suction, suction	29 (76)	7 (58)	0.28
Number of punctures	3 (2–7)	3 (2–4)	0.18
Endoscopist, expert	10 (26)	5 (42)	0.47
Rapid on‐site evaluation	1 (3)	2 (17)	0.14
Tumor cellularity ≥20% on pre‐check by pathologists	37 (97)	5 (42)	<0.01
Image analysis by ImageJ			
Tumor area content, %	18.5 (0.6–37.2)	10.5 (0.0–58.8)	0.29
Tissue area, mm^2^	8.1 (0.1–49.8)	6.7 (1.5–14.3)	0.16
Tumor area content ≥20%	16 (42)	3 (25)	0.33
Tissue area ≥16 mm^2^	9 (24)	0	0.09
Tissue area ≥25 mm^2^	4 (11)	0	0.56
Tissue area ≥16 mm^2^ and tumor area content ≥ 20%	4 (11)	0 (0)	0.56

*Note*: Numbers are shown in *n* (%) or median (range).

Abbreviation: CGP, comprehensive genomic profiling.

## DISCUSSION

In our retrospective study, CGP tests attempted by EUS‐TA samples using a 22‐gauge Franseen needle were successful in 76%, which was comparable to those of previous studies using a 19‐gauge Franseen needle.^24^, ^25^ The Safety profile of EUS‐TA was acceptable with 4% of all AEs and null serious AEs. In the image analysis using ImageJ, the tumor content and tissue area did not necessarily fulfill the criteria for CGP even in cases with successful CGP tests.

Due to the advancement of new biopsy needles,^29^, ^30^ the use of 19‐gauge needles for EUS‐TA only for pathological diagnoses has been decreased in clinical practice. The major disadvantage of 19‐gauge needles is their poor maneuverability. Previous studies showed inferior diagnostic yield of 19‐gauge needles, especially in cases with pancreas head or uncinate lesions^31^, ^32^, ^33^ but we have seen the resurgence of 19‐gauge needles in this era of CGP tests.^24^, ^25^ Given the increasing utilization of CGP tests in many centers, technical difficulties of 19‐gauge needles might be encountered in non‐high volume centers. Our study results suggested that a 22‐gauge Franseen needle, which is one of the current standard‐of‐care needles for EUS‐TA, is also feasible for CGP tests with a success rate of 76%. Furthermore, the safety of 19‐gauge needles is also of concern with a reported AE rate of 9.0%. The AE rate was 4.0% in our study; one mild pancreatitis and one mild hemorrhage, which is acceptably low but still seems higher than that in previous studies of 22‐gauge FNB needles.^18^ Of note, the median number of passes was three as compared to two in our previous multicenter study.^18^ As previously reported,^34^, ^35^ two passes of 22‐gauge FNB are sufficient for pathological diagnoses. Thus, it is possible that one extra pass was taken considering the possibility of CGP test submission and might contribute to the slightly higher AE rate. Bang et al. recently reported two dedicated passes 22‐gauge Franseen needles can provide optimal specimen for genome profiling,^36^ but the sample size is only 33 cases in total. One retrospective study suggested the number of passes was associated with successful CGP tests in 22‐gauge needles, but not in 19‐gauge needles.^37^ The appropriate number of passes both for 19‐ and 22‐gauge needles should be further evaluated in the context of CGP tests.

Exploratory analyses for successful CGP tests suggested the use of suction and non‐pancreatic cancer as potential prognostic factors. The slow pull technique is recommended for Franseen needles^38^ in terms of the pathological diagnosis of pancreatic tumors but the appropriate suction method in terms of CGP test may differ. In general, the application of suction might hinder the pathological diagnosis due to the increased blood contamination, but it may not interfere with CGP tests. In our study, the slow pull technique was applied only in two cases and further evaluation of the appropriate suction method is needed.

In our analyses, a tissue area of ≥16 mm^2^ on ImageJ was not a predictive factor for successful CGP tests, but when a tissue area was ≥16 mm^2^ on ImageJ, the CGP test was successful in all cases. Of note, the tumor content by Image J reflected the tumor area, not tumor cell content, which is also crucial for CGP test suitability. In clinical practice, pathologists typically conduct visual assessments,^39^ of which differ from the quantitative method used in this study with ImageJ. Since it's easier to calculate tumor area content compared to tumor cell content by counting tumor cell nuclei, tumor area content as well as tissue area might be useful to ensure proper tissue acquisition for CGP tests. Further examination with large sample size is mandatory to confirm the utility of ImageJ for CGP tests in clinical practice.

There are some limitations in this study. The single‐center, retrospective design is the major limitation. The criteria of 1st QC check are semi‐quantitative and highly dependent on pathologists at each center. Technically, the submission of additional thin slices to CGP tests might increase the success rates of CGP tests, which are also differently performed by each center. Thus, our study results need external validation. Secondly, a successful CGP test does not necessarily mean the quality and quantity of the tumor genome are ideal. We sometimes encounter tumor cell content that is not high enough and the results of tumor mutational burden and microsatellite instability are shown for references only. Similarly, the DNA yield also included both tumor and non‐tumor‐derived DNA. Thus, we need the common criteria for the evaluation of EUS‐TA specimens for CGP tests to allow inter‐study comparison. Although we employed the image analysis by ImageJ for evaluation of the tissue area and tumor area content, ultimately we need to count the number of tumor cells as surrogates of the DNA or RNA yield from the tumor during EUS‐TA procedures. Further investigations are warranted to develop an on‐site evaluation system for CGP tests, similar to ROSE or macroscopic onsite evaluation.^40^, ^41^, ^42^

In conclusion, the present study suggested the feasibility of CGP tests using a 22‐gauge Franseen needle. To further confirm our hypothesis, we are planning a randomized controlled trial to show the non‐inferiority of 22‐gauge to 19‐gauge FNB needles for CGP tests.

## CONFLICT OF INTEREST STATEMENT

Yousuke Nakai is an Associate Editor of Digestive Endoscopy. YN received a research grant from Boston Scientific Japan, Fujifilm Corporation, and honoraria from Boston Scientific Japan, Fujifilm Corporation, and Olympus Corporation. MF received a research grant from Olympus Corporation, Fujifilm Corporation, and honoraria from Fujifilm Corporation, Olympus Corporation.

## Supporting information

TABLE S1 Details of treatment based on genetic abnormalities.
