# Supplementary material for: Feasibility of comprehensive genomic profiling using endoscopic ultrasound‐guided tissue acquisition with a 22‐gauge Franseen needle
Source: DEN Open. 2024 Apr 15;4(1):e365. doi: 10.1002/deo2.365 (PMC11019146; doi:10.1002/deo2.365)
Supplement: Supplementary file 1 — TABLE S1 Details of treatment based on genetic abnormalities. [file DEO2-4-e365-s001.docx]

**Supplementary Table 1. Details of treatment based on genetic abnormalities**

| Case | Age | Sex | CGP | Site of cancer | Result | Treatment attainment |
| --- | --- | --- | --- | --- | --- | --- |
| 1 | 74 | M | F1　Liquid* | Pancreatic cancer | *ERBB2* | N (No matched clinical trial) |
| 2 | 59 | M | NOP | Pancreatic cancer | *KRAS G12C* | Y (Clinical trial) |
| 3 | 70 | M | NOP | Pancreatic cancer | *MSH6* | N (No matched clinical trial) |
| 4 | 63 | F | NOP | Pancreatic cancer | *BRCA2* (somatic + germline mutation) | Y (PARP inhibitor) |
| 5 | 84 | M | NOP | Pancreatic cancer derived from IPMN | *BRAF V600_K601delinsE* | N (Ongoing standard treatment) |
| 6 | 88 | M | NOP | Hilar cholangiocarcinoma | *ERBB2 amplification* | N (Poor general condition) |
| 7 | 81 | M | NOP | Gallbladder cancer | *ATM* (germline mutation) | Y (Cisplatin) |
| 8 | 73 | M | NOP | Combined HCC and cholangiocarcinoma | TMB-H (18.6/Mb) | N (Poor general condition) |
| 9 | 73 | F | NOP | NEC | *BRAF K601E* | N (Poor general condition) |
| 10 | 87 | M | NOP | CUP | TMB-H (10.9/Mb) | Y (Nivolumab) |

*After failed NOP. CGP, comprehensive genomic profiling; CUP, cancer of unknown primary; NOP, OncoGuide™ NCC Oncopanel System; F1 liquid, FoundationOne^®^ Liquid CDx; HCC, hepatocellular carcinoma; IPMN, intraductal papillary mucinous neoplasm; NEC, neuroendocrine carcinoma; TMB-H, tumor mutational burden-high; PARP, poly ADP-ribose polymerase.
